# Abnormalities of the Optic Nerve in Down Syndrome and Associations With Visual Acuity

**DOI:** 10.3389/fneur.2019.00633

**Published:** 2019-06-14

**Authors:** Lavinia Postolache

**Affiliations:** Queen Fabiola University Children's Hospital, Université Libre de Bruxelles, Brussels, Belgium

**Keywords:** Down syndrome, optic nerve, visual acuity, optic disc size, optic disc drusen, physiological cup, tilted disc

## Abstract

**Background:** Various ocular anomalies are present in the vast majority of individuals with Down syndrome; however, we know little about optic nerve abnormalities. The aim of this cross-sectional comparative study was to describe optic disc morphology in patients with Down syndrome and to determine if the differences found are potentially related to visual acuity.

**Subjects/methods:** Assessable fundus images were obtained in 50 children with Down syndrome and 52 children without Down syndrome. Morphological analysis of the optic nerve was performed, including the disc-to-macula distance (DM) to disc diameter (DD) ratio (DM/DD), the cup-to-disc ratio and optic disc ovality. Data relating to ophthalmological status were retrospectively analyzed to evaluate the possible causes of reduced visual acuity.

**Results:** DM/DD was significantly larger (*p* = 0.0036) and the cup-to-disc ratio was significantly smaller (*p* = 0.018) in children with Down syndrome, compared to controls. The optic discs were also more frequently torted (*p* = 0.034), tilted (*p* = 0.0049) and oval (*p* = 0.026). Furthermore, crescents (*p* = 0.0002), peripapillary atrophy (*p* = 0.0009), and pigment anomalies (*p* < 0.0001) were also more prevalent in children with Down syndrome than in those without. Visual acuity was significantly lower in children with Down syndrome compared to controls with similar refraction problems and strabismus prevalence (*p* < 0.0001). The mean DM/DD and the presence of a crescent was not directly related to visual acuity (*r* = 0.39, *p* = 0.31), (*r* = 0.35, *p* = 0.12) respectively. Visual acuity was diminished in 80% of children with Down syndrome and the smallest discs and in 84% of those with tilted discs. However, other causes may contribute to the diminished visual acuity in these cases.

**Conclusion:** The optic nerve head in children with Down syndrome is affected by various anatomical and developmental abnormalities. Unrelated to refraction (spherical equivalent), the optic discs appear smaller and more frequently mal-inserted in Down syndrome. Optic disc hypoplasia, as well as severe tilting, may reduce vision but they do not represent major contributors to the decrease of vision in such children. As these children often have multiple ocular and neurosensory problems, it remains challenging to relate visual acuity problems with a specific abnormality. Smaller discs may lead to optic disc drusen formation in children with Down syndrome.

## Introduction

An increased prevalence of ocular abnormalities has been observed in patients with Down syndrome over the last century. Some of these anomalies, such as slanting fissures, epicanthal folds (1), Brushfield spots and peripheral iris thinning with fewer contraction furrows (2–4) have no impact upon visual acuity. Others, such as significant refractive errors, strabismus with amblyopia (5), nystagmus (6, 7), hypoaccommodation (8), and cataracts (5), have been the subject of numerous publications and are considered to be some of the key factors responsible for the diminished visual acuity in children with Down syndrome. In contrast, reports relating to retinal and optic nerve anomalies in cases of Down syndrome are less frequent and the functional impact of these conditions remains poorly defined (9). The most consistent finding, with regards to the optic nerve, is related to an increased number of vessels crossing the optic disc margin (10–12). Other optic nerve anomalies, such as hypoplasia, elevation, pallor, crescents, tilted discs, peripapillary atrophy, and pigment anomalies, have been described sporadically in some studies and case reports (3, 13–24). Only one study systematically analyzed the optic nerve appearance in a large series of children with Down syndrome and found an anomalous optic nerve in 14%, based on medical charts (25). Contrariwise, other studies reported none (5) or <5% of such abnormalities (19, 22). In most instances, no direct relation to visual acuity was described.

However, even in children with Down syndrome with no apparent ocular anomalies, performance in visual acuity tests is often lower compared to their peers without Down syndrome (6). In addition, contrast sensitivity (25) and visual evoked potentials (26) have been found to be abnormal in children with Down syndrome; this finding is indicative of some form of neurosensory deficit. This neurosensory deficit could be partially explained by the structure of the brain, as individuals with Down syndrome exhibit a global reduction in volume, fewer neurons and abnormal synapses (27, 28).

The present study aimed to use fundus imaging to investigate the optic nerve head in children with Down syndrome compared to controls and strived to facilitate a better understanding of the literature discrepancies on the subject. As both brain and other eye segments are frequently different in Down syndrome, we hypothesize that the optic nerve is also anomalous, and such anomalies may reduce the visual acuity in these children.

Better recognizing the potential optic nerve anomalies in individuals with Down syndrome, may help to identify indicators for their reduced visual acuity. Knowledge of such information would also improve their medical care.

## Materials and Methods

### Type of Study

This was a cross-sectional, non-interventional, comparative study of children with Down syndrome and controls.

### Subjects and Setting

During a 5-year period (June 2013—November 2018), 64 patients with Down syndrome underwent complete ophthalmological examination, including fundus imaging at Queen Fabiola University Children's Hospital in Brussels, Belgium. In 14 of these patients, images were of poor quality; consequently, these 14 patients were excluded from the study. The control group featured 52 non-hospitalized children without Down syndrome, who had been examined for refractive or strabismus issues in 2018 and had undergone similar fundus imaging.

### Informed Consent

We obtained written informed consent from all children and their parents. The Institutional Review Board, and Institutional Ethics Committee, of Queen Fabiola University Children's Hospital, also provided their approval for this study to take place (CHE n°23/19). All examinations were performed in accordance with the principles and tenets of the Declaration of Helsinki.

### Ophthalmologic Examinations

All children underwent cycloplegic autorefraction, orthoptic evaluation, anterior segment biomicroscopy and posterior segment assessment, by both indirect ophthalmoscopy and fundus imaging. Myopia was defined as < -0.75 diopters spherical equivalent and hyperopia as > +0.75 diopters spherical equivalent. Clinically significant astigmatism was considered if the cylinder value was >1.00 diopter (plus cylinder) and classified as “with the rule” and “against the rule” if the axis was at 90 and 180° meridian, respectively. The oblique axis was considered between 10–80° and 100–170°. Anisometropia was defined as a difference in spherical equivalent, or in astigmatism, of >1 diopter between the two eyes. Orthoptic examinations were used to assess ocular motility and the presence of strabismus and nystagmus. The accommodation was evaluated by dynamic retinoscopy, but only in children with Down syndrome.

The visual acuity of children was investigated in children with Down syndrome and compared to controls; prior to comparison, the data were adjusted for age and cognitive status.

### Imaging

Fundus imaging was performed using a non-mydriatic fundus camera (Visucam R 500, Zeiss, Jena, Germany). This device featured a telecentric optical system to adjust measurements based on refractive errors and provided good levels of focus.

### Assessment of Images

Optic disc images were defined as assessable if the optic disc margin and fovea could be clearly identified. Based on the fundus photographs, image analysis was carried out to determine a range of optic nerve variables, as described in Table 1.

**Table 1:** Optic nerve variables systematically analyzed.

**Variable**		**Definition/Criteria of analysis**
Disc-to-macula distance (DM) to disc diameter (DD) ratio (DM/DD)		DM/DD indicates the number of optic discs that can be apposed between the fovea and the center of the disc (29). Figure 1A shows how DM/DD was calculated.
Double ring sign		An outer ring formed by the area composed of bare sclera and an inner ring formed by the retinal nerve fibers (30)
Physiological cupping		Presence or absence
Cup-to-disc ratio		Horizontal cup diameter-to-horizontal disc diameter ratio
Optic disc ovality		Vertical-to-horizontal disc diameter ratio (or the maximal-to-minimal disc diameter ratio if the vertical diameter was oblique) (31). Oval discs were defined as having an ovality ratio >1.33
Torted optic disc		Deviation of the long axis >15°From the vertical meridian (32)
Peripapillary crescents		Presence or absence
Peripapillary crescents^a^	Scleral crescent	If the sclera was visible in the crescent while the choroid and the pigment epithelium did not reach the optic disc margin (33)
	Choroidal crescent	If the choroid was visible in the crescent, as only the pigment epithelium did not reach the optic disc margin (34).
Peripapillary crescents localization		Temporal Below the disc (if the wider area of the crescent was inferiorly located) Annular (if the crescent was present in at least three quadrants), Other (for all other localizations).
Peripapillary atrophy		Atrophy is recognized by less well-defined contour compared to crescents, and variable degrees of extension.
Intra and peripapillary pigment anomalies	Gray crescents	An extension of the retinal pigment epithelium and Bruch membrane within the peripheral tissue of the optic disc (35).
	Conus pigmentosum^b^	Dark pigmented zone, resulting from localized proliferation of retinal pigment epithelium (36).
	Intrapapillary pigment	Papillary involvement of uveal melanocytes (37)
Tilted disc^c^		If the optic nerve head was tilted in its sagittal axis.
Color		Graded as normal, temporal pallor or overall pallor.
Contour		Graded as sharp or elevated.

*Definition and criteria of analysis*.

a *Both crescents are sharply boarded, and they were differentiated on photographic images because the scleral crescent was whitish and the choroidal was gray*.

b *The conus pigmentosum was differentiated from the choroidal crescent as it was more darkly pigmented, usually with a less well-defined contour and various shapes*.

c *The fundus image was corroborated by ophthalmoscopic examination to allow a better three-dimensional visualization of the optic nerve head insertion in confirming the tilted disc*.

**Figure 1:**
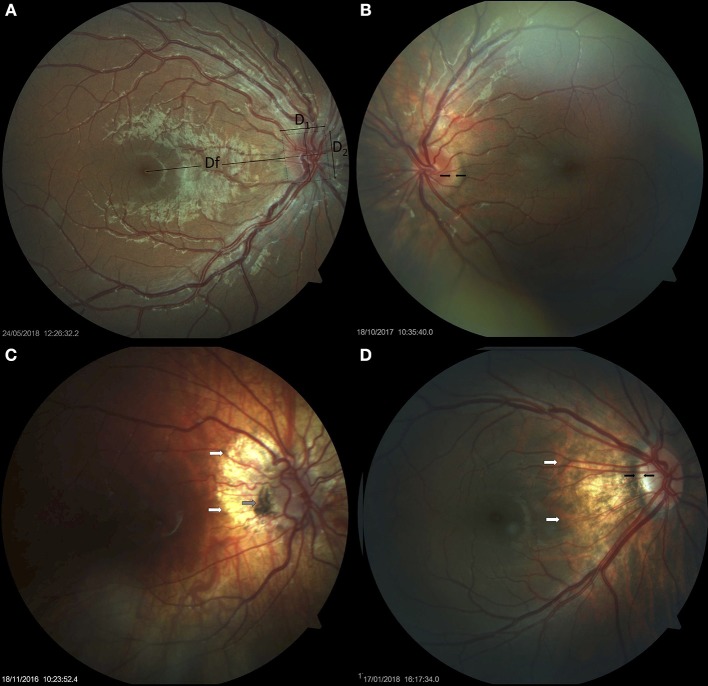
Small optic discs in Down syndrome. **(A)** Small optic disc with vascular tortuosity in a child with Down syndrome. This image exemplifies the formula used in the estimation of the disc-to-macula distance (DM) to disc diameter (DD) ratio (DM/DD): Dfx2+ D_1_/D_1_+ D_2_ (38, 39)_._ Both vertical and horizontal disc diameters were considered, to compensate for oval discs. **(B)** Small, round optic disc with a double ring sign between the black arrows. **(C)** Hypoplastic disc of a child with Down syndrome. Papillary vascular malformation is evident. A large halo of peripapillary atrophy is seen at 360° (white arrows). The gray arrow indicates an area of pigmented epithelium hypertrophy at the temporal margin of the disc. **(D)** Small tilted optic disc in a child with Down syndrome and myopia. A scleral crescent is visible at the temporal margin (between the black arrows). The disc is oval and bean-shaped in this case, with a hyperpigmented halo. An extensive area of peripapillary atrophy, with visible choroidal vessels, is evident (white arrows).

### Statistical Analysis

Results are expressed as mean and standard deviation (SD) for quantitative variables. Frequency tables (number, percentage) were used for categorical variables. Mean values of subject-related variables (for example, age) were compared using Student *t*-test while the chi-square test was used to compare proportions (for example, gender). For quantitative eye-related measurements (for example, visual acuity), the Down syndrome and control groups were compared using mixed-effects models to account for within-subject variability between the two eyes. For binary or categorical findings (for example, the presence of crescents), a generalized linear mixed model (logistic, ordinal logistic or multinomial) was used to test for differences between subjects with Down syndrome and controls in order to account for left and right eye assessments. To study the relationship between eye visual acuity and other characteristics, such as DM/DD, data were combined from both eyes and analyzed by multivariate canonical correlation analysis. This method permits calculation of the “best” correlation between a weighted sum of visual acuity for the left and right eye, on the one hand, and a weighted sum of DM/DD for the left and right eye, on the other hand; this relationship was assessed using the first canonical correlation. All results were considered to be statistically significant at the 5% level (*p* < 0.05). Calculations were consistently performed on all available data; missing values were not replaced nor imputed. Statistical analyses were performed using SAS version 9.4 (SAS Institute, Cary, NC, USA) and R version 3.5 (R Foundation for Statistical Computing, Vienna, Austria) software.

## Results

### Study Demographics

The characteristics of the study population are summarized in Table 2. The groups were globally identical, with the exception that the children with Down syndrome were older than the controls.

**Table 2:** Demographic data of children with Down syndrome and controls.

**Variable**		**Down syndrome**	**Controls**	***P*-value**
Number		50	52	
Age (years)		9.8 ± 3.8	7.6 ± 3.0	0.0016
Sex	Boys	29 (58.0)	23 (44.2)	0.16
	Girls	21 (42.0)	27 (55.8)	
Origin	White	42 (84.0)	45 (86.5)	0.72
	Black	8 (16.0)	7 (13.5)	

### Refractive and Orthoptic Problems

**Figure 2:**
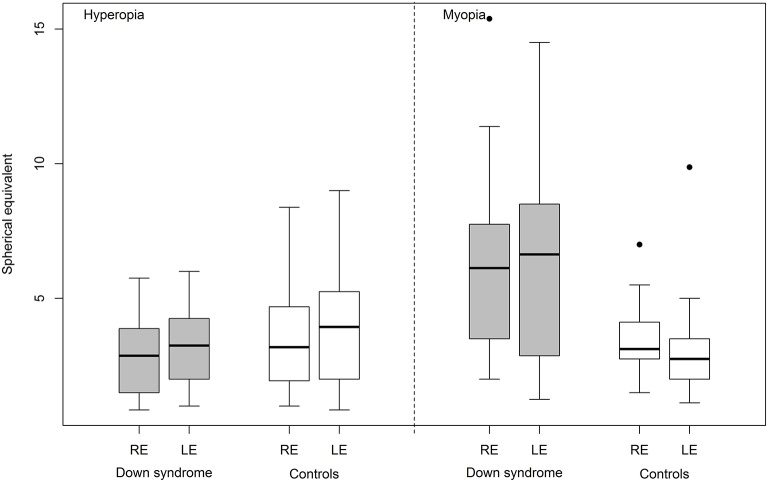
Hyperopia and myopia (spherical equivalent) in Down syndrome vs. control (RE, right eye; LE, left eye).

No significant differences were found between the two groups in relation to spherical equivalent (Figure 2), strabismus or anisometropia. Astigmatism (unilateral or bilateral) was identified in 29 (58%) children with Down syndrome and 23 (44%) controls. Only the proportion of cases with oblique astigmatism was significantly different in Down syndrome than in controls (42 vs. 12%, *p* = 0.0011).

Strabismus was identified in 25 (50%) children with Down syndrome and 21 (40.4%) controls (*p* = 0.33) while anisometropia was identified in 15 (30%) children with Down syndrome and 21 (40.4%) controls (*p* = 0.27). Horizontal nystagmus (latent or manifest) was present in 15 (30%) children with Down syndrome but was not detected in any of the controls (*p* < 0.001). Accommodative status was determined in 42 children with Down syndrome; hypoaccommodation was identified in more than half of these children (59.5%).

Forty-seven (94%) children with Down syndrome and forty-five (86.5 %) controls had a form of optical correction; over 80% of subjects were compliant in terms of the use of the optical correction devices. To counteract their accommodative problems, 23 children with Down syndrome used bifocal or multifocal lenses.

### Anterior Segment Anomalies

Four children with Down syndrome and none of the controls had anterior segment anomalies (three children had partial cataracts and one child had a unilateral corneal scar), which may have influenced visual acuity.

### Posterior Segment Anomalies

#### Fundus Anomalies

Myopic fundus was identified in eight children with Down syndrome and in two controls. No other retinal anomalies that were likely to reduce vision were noted in children with Down syndrome or the controls.

#### Optic Nerve Anomalies

Overall, children with Down syndrome had a larger DM/DD (*p* = 0.0036) and a smaller cup-to-disc ratio (*p* = 0.018) than controls (Table 3).

**Table 3:** DM/DD, absence of physiological cupping and cup-to-disc ratio in children with Down syndrome vs. controls.

**Parameter**	**Down syndrome**		**Controls**		***P*-value**
	**Right eye**	**Left eye**	**Right eye**	**Left eye**	
DM/DD	3.0 ± 0.45 (*n* = 47) ^a^	3.0 ± 0.44 (*n* = 44)^a^	2.8 ± 0.31 (*n* = 52)	2.8 ± 0.3 (*N* = 52)	0.0036
Absence of physiological cup	26 (52%) (*n* = 50)	23 (46%) (*n* = 50)	19 (36.5%) (*n* = 52)	21 (40.4%) (*n* = 52)	0.25
Cup-to-disc ratio	0.2 ± 0.13 (*n* = 24)^b^	0.2 ± 0.12 (*n* = 27)^b^	0.3 ± 0.15 (*n* = 33)^b^	0.3 ± 0.14 (*n* = 31)^b^	0.018

*DM/DD: disc-to-macula distance (DM) to disc diameter (DD) ratio*.

a *DM/DD was unavailable for six patients (unilateral/bilateral), caused by the impossibility to precisely determine the disc margin or the foveal reflex*.

b *Horizontal cup diameter-to-horizontal disc diameter ratio, calculated for those with physiological cupping*.

**Figure 3:**
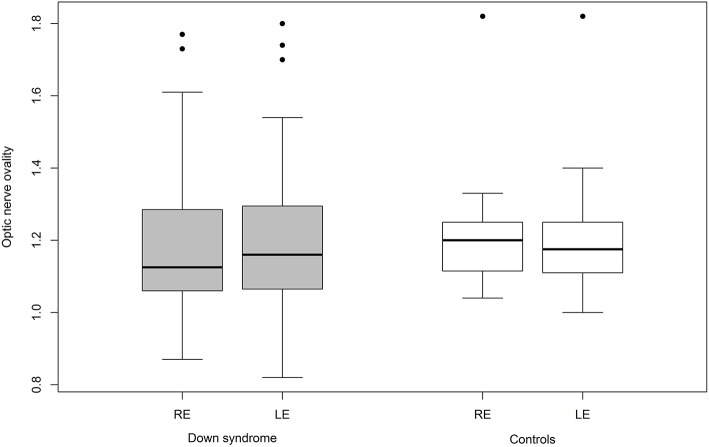
Optic disc ovality in Down syndrome vs. control (RE, right eye; LE, left eye).

Mean ovality was not significantly different when compared between the two groups (Table 4). However, 12 children with Down syndrome (25%) had an oval optic nerve (six bilateral and six unilateral) compared to only five controls (9.6%; one bilateral and four unilateral; *p* = 0.026) (Figure 3).

**Table 4:** Optic disc ovality and torsion and the presence of crescents, peripapillary atrophy and pigment anomalies in children with Down syndrome vs. controls.

**Parameter**	**Down syndrome**		**Controls**		***P*-value**
	**Right eye**	**Left eye**	**Right eye**	**Left eye**	
Optic disc ovality	1.2 ± 0.20 (*n* = 48)^a^	1.2 ± 0.22 (*n* = 47)^a^	1.2 ± 0.12 (*n* = 52)	1.2 ±0.13 (*n* = 52)	0.92
Optic disc torsion	16 (32%) (*n* = 50)	12 (24%) (*n* = 50)	2 (3.8%) (*n* = 52)	6 (11.5%) (*n* = 52)	0.034
Crescents present	21(42%) (*n* = 50)	21(42%) (*n* = 50)	5 (9.6%) (*n* = 52)	5 (9.6%) (*n* = 52)	0.0002
Peripapillary atrophy	18 (36%) (*n* = 50)	17 (34%) (*n* = 50)	3 (5.8%) (*n* = 52)	3 (5.8%) (*n* = 52)	<0.05
Peripapillary pigment anomalies	17 (34%) (*n* = 50)	17 (34%) (*n* = 50)	2 (3.8%) (*n* = 52)	2 (3.8) (*n* = 52)	<0.0001

a *The vertical-to-horizontal disc diameter ratio was unavailable for five eyes of children with Down syndrome with overall elevated contour, making it impossible to differentiate the disc margins in a precise manner*.

**Figure 4:**
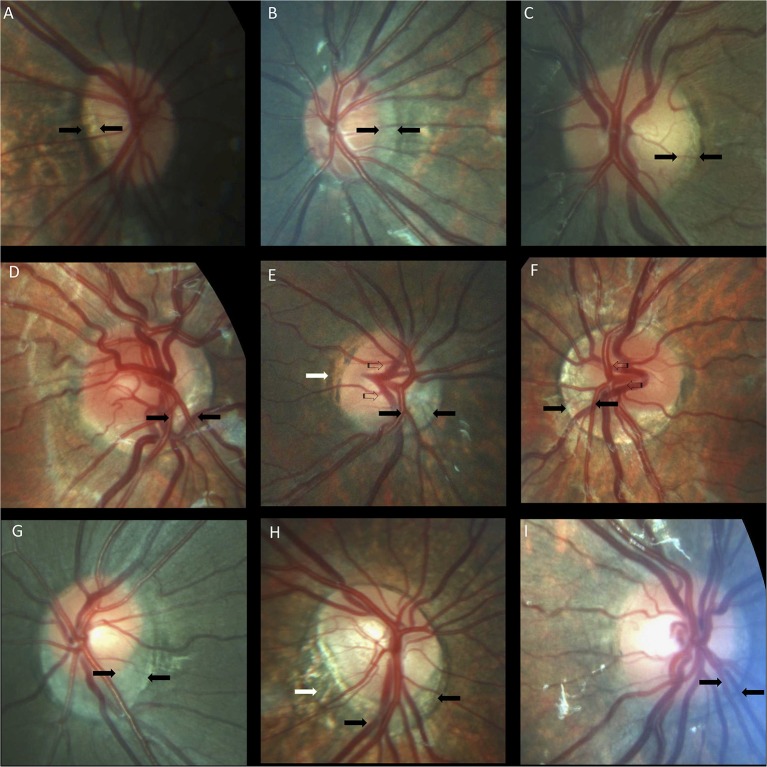
Optic nerve crescents in children with Down syndrome. **(A)** Oval and tilted optic disc with a temporal crescent (black arrows) in a child with Down syndrome and myopia. **(B)** Choroidal crescent located temporally (black arrows) in a small, tilted disc from a child with Down syndrome and high myopia. **(C)** Small temporal crescent (black arrows) in a child with Down syndrome and hyperopia. **(D)** Small, tilted disc with vascular tortuosity. A scleral crescent is located below the disc and extends nasally (black arrows). **(E)** Tilted disc with situs inversus of the vessels (striped arrows). A large choroidal crescent is evident below the disc and extending into the nasal area (between the black arrows). Peripapillary atrophy is noted at the temporal margin of the disc (white arrows). **(F)** Tilted disc in which the scleral crescent, although wider below the disc, takes an annular form. Situs inversus, in which the vessels emerge nasally, is also evident (striped arrows). **(G)** Choroidal crescent, located below the disc with inferonasal and temporal extension (black arrows), in a child with Down syndrome and hyperopia. The disc appears equally tilted in this case. **(H)** Tilted and torted optic disc of a child with Down syndrome with myopic astigmatism. A choroidal crescent is evident below the disc (black arrows) along with a large zone of temporal peripapillary atrophy (white arrow). Note the bean-shaped optic disc in this case. **(I)** A smaller choroidal crescent, located below the disc and nasally, in a child with Down syndrome and hyperopia. In the upper and central rows, the optic discs have no physiological cupping.

**Figure 5:**
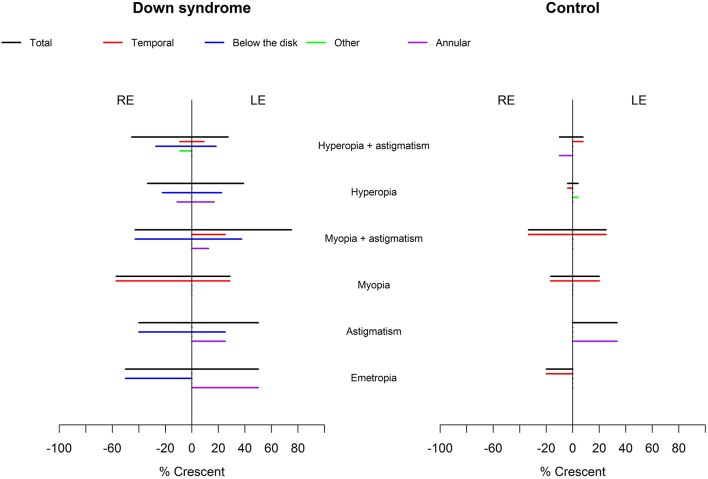
Relationship between crescent localization and refraction in children with Down syndrome vs. controls. Crescents (black lines) were evident in children with Down syndrome, without association with a specific refractive status. In controls, the crescents were more prevalent in those with myopia and astigmatism. Temporal crescents (red lines) were evident mostly in children with and without Down syndrome with myopia and myopic astigmatism. Crescents below the disc and annular crescents were more prevalent in children with Down syndrome (blue and purple lines). Other localizations (green lines) were rare.

Crescents were found in 24 (48%) children with Down syndrome (six unilateral and 18 bilateral) and in only six (11.5%) controls (two unilateral and four bilateral). Scleral crescents were evident in four children with Down syndrome (one unilateral and three bilateral) and in three controls (all bilateral). Choroidal crescents were present in 24 (48%) children with Down syndrome (six unilateral and 18 bilateral) and in only six (11.5%) controls (two unilateral and four bilateral) (*p* = 0.043). Figure 4 illustrates the different types and localizations of the crescents found in children with Down syndrome. The relationship between crescent localization and refractive status is illustrated in Figure 5.

**Figure 6:**
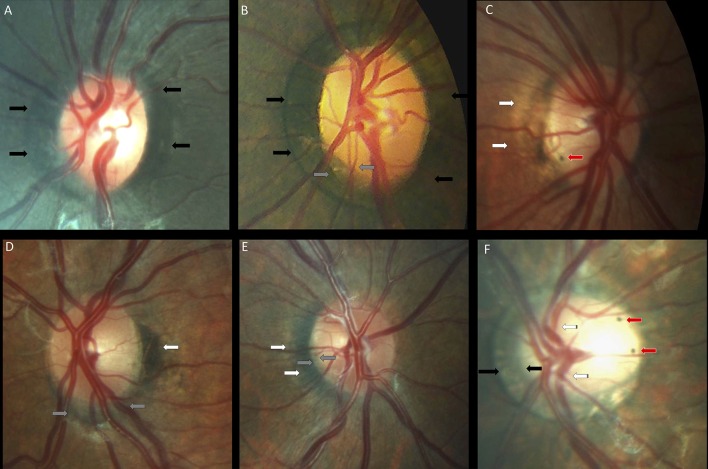
Peripapillary pigmentary anomalies in children with Down syndrome. **(A,B)** Failure of the pigment epithelium to reach the optic disc margin for 360° of the optic disc margin in two children with Down syndrome (black arrows). **(B)** Inferotemporal small gray crescent (between the gray arrows). **(C)** Small intrapapillary pigment dot (red arrow) in an optic disc with temporal peripapillary atrophy (white arrows). **(D)** Choroidal crescent (gray arrows) below the disc with temporal pigment epithelium hypertrophy (white arrows). **(E)** Temporally-located conus pigmentosum (white arrows). The pigment extends into the optic disc substance, creating the appearance of a small gray crescent (gray arrows). **(F)** A tilted disc with an annular crescent (black arrows) and situs inversus of the vessels (striped arrows) in a child with Down syndrome and no refraction error. Two intrapapillary pigment dots are noted on the temporal side of the disc (red arrows).

Peripapillary and papillary pigment anomalies were found in 15 (30%) children with Down syndrome, and in one control, and consisted mainly of peripapillary pigment epithelium proliferation of various different shapes (for example, conus, ring, and plaque). Gray crescents and intrapapillary intense pigmentation were found sporadically. Figure 6 shows various different types of pigment anomalies found in children with Down syndrome.

**Figure 7:**
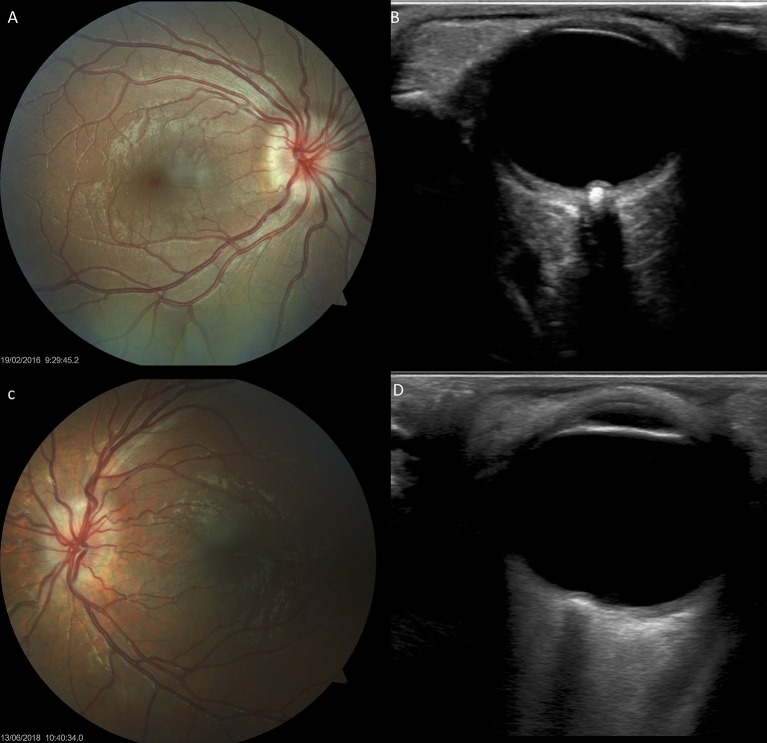
Optic disc drusen in children with Down syndrome, as evidenced by fundus imaging and ocular B-scan ultrasound. **(A,C)** Optic disc drusen in two children with Down syndrome; a spoke-like vessel pattern is evident in **(A)**. The optic discs appear smaller, with no physiological cupping. **(B,D)** Ocular B-scan ultrasound in the same patients, showing ovoid echogenic lesions, with acoustic shadow, at the junction of the retina and the optic nerve.

Thirteen (26%) children with Down syndrome, and two controls (3.8%), had a tilted optic disc (four unilateral and 11 bilateral) (*p* = 0.0049). Examples of tilted discs are shown in Figures 1D, 4A–H, 6F. No significant difference was evident with regard to the color or contour of the optic nerve in children with Down syndrome when compared to the controls. However, four (8%) children with Down syndrome had optic disc drusen; this condition was confirmed by B-scan ultrasound (Figure 7).

### Visual Acuity

**Figure 8:**
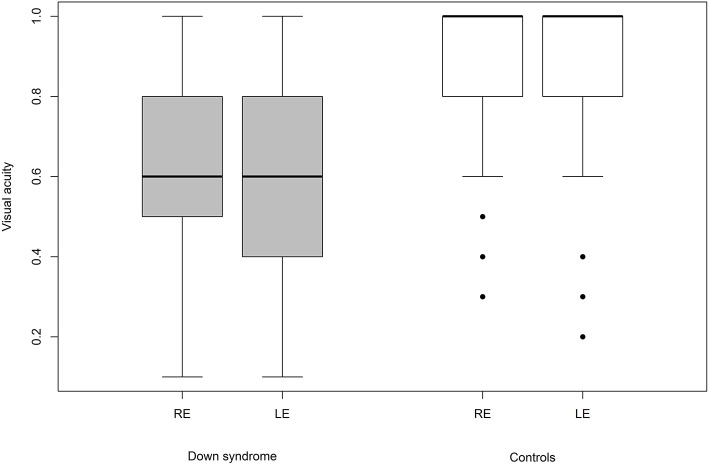
Visual acuity in children with Down syndrome without opacities of the media vs. controls.

**Figure 9:**
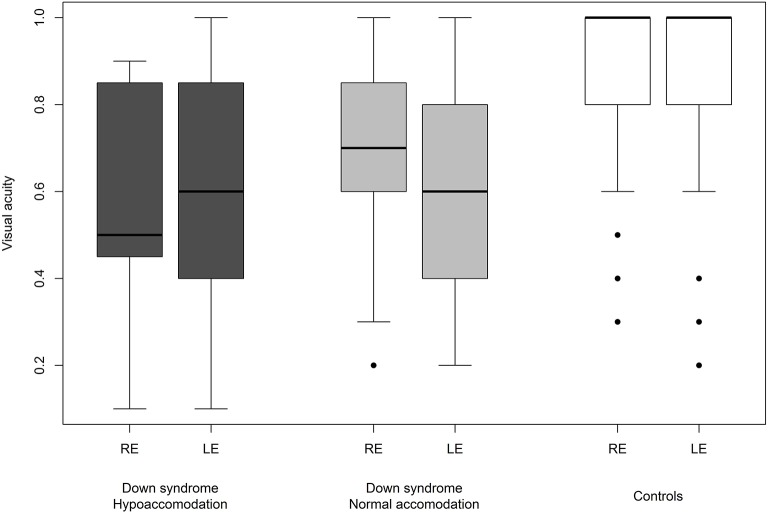
Visual acuity in children with Down syndrome and hypoaccommodation compared to those with normal accommodation and to controls.

**Figure 10:**
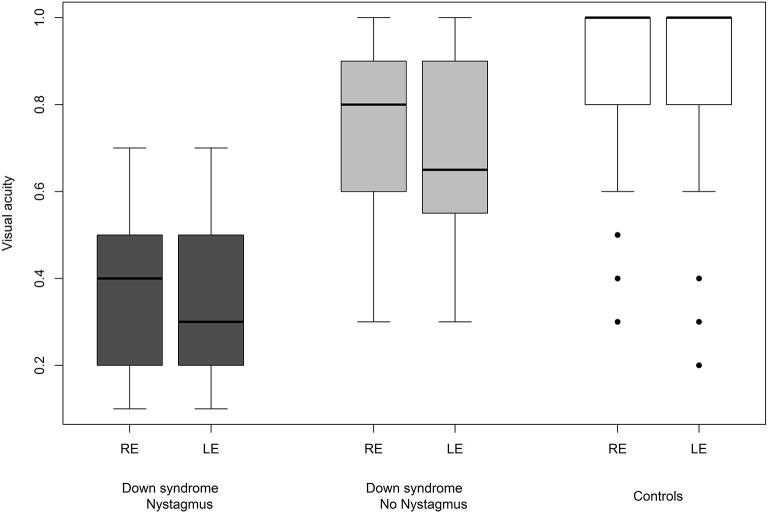
Visual acuity in children with Down syndrome with and without nystagmus and in controls.

We were able to successfully measure the visual acuity in 41 (82%) children with Down syndrome and in all of the controls. Overall, the visual acuity was worse in children with Down syndrome, even if those with partial cataracts or corneal scars were excluded (Figure 8). We failed to detect any sign of a significant relationship between hypoaccommodation and visual acuity (*p* = 0.10; Figure 9). The presence of crescents did not have any significant influence on the visual acuity of children with Down syndrome (*r* = 0.35, *p* = 0.12). Furthermore, there was no association between DM/DD and visual acuity (*r* = 0.39, *p* = 0.31). Interestingly, visual acuity was significantly lower in children with Down syndrome plus nystagmus than in children with Down syndrome without nystagmus (*p* = 0.0001; Figure 10).

## Discussion

In the present study, careful morphological assessment of the optic nerve head permitted the detection of various differences when comparing children with Down syndrome and controls. We also investigated several potential causes of reduced vision in children with Down syndrome.

### Optic Disc Size

DM/DD provides an objective estimation of the optic nerve head size and eliminates the magnification of high refractive errors (38, 40), like myopia which could lead to an underestimation of the disc hypoplasia. A DM/DD >4 was established as being reliably supportive for the diagnosis of optic nerve hypoplasia, while a DM/DD >3.5 was suggestive of mild hypoplasia (29, 40–42). Other signs that are considered to be supportive for the diagnosis of optic nerve hypoplasia are vascular tortuosity, a double ring sign and the absence of a physiological cup. Without cupping, the optic discs are known to be smaller than average; the size of the scleral canal influences not only the presence, but also the depth of the physiological cup (33, 43).

There are few reports relating to the presence of optic nerve hypoplasia in individuals with Down syndrome in the existing literature (14, 44–46). One of these studies reported the presence of optic nerve hypoplasia in 10% of children with Down syndrome, based on the presence of a double ring sign (13). In the present study, children with Down syndrome had a mean DM/DD that was significantly higher than that in the controls (*p* = 0.036), indicating that these subjects had smaller optic discs. Nine (18%) children with Down syndrome and one control had a DM/DD >3.5. In half of these subjects, the disc was also tilted. A double ring sign was observed in four (8%) children with Down syndrome; three of these also had a DM/DD >3.5. Figure 1 illustrates examples of small optic discs in children with Down syndrome. A physiological cup was absent in 22 (44%) children with Down syndrome and 17 (32.7%) controls. The prevalence of discs without physiological cupping was higher than that previously reported for healthy children (12 %) (47); we believe that this was because many of the children in the present study had hyperopia. Examples of optic discs without a physiological cup are shown in Figures 1, 4A–F, 7A,C. Furthermore, the children with Down syndrome had a cup-to-disc ratio that was significantly smaller when compared to controls (*p* = 0.018).

Although the children with Down syndrome had a smaller disc than the controls, the diagnosis of optic disc hypoplasia remains difficult. To diagnose optic nerve hypoplasia, evidence regarding the size of the disc should be reinforced with evidence of reduced vision and visual field defects (38). In children with Down syndrome, a reliable assessment of both visual acuity and visual field is particularly challenging. Moreover, the visual acuity of these children can be reduced by a range of different causes, which may vary between subjects.

Optic nerve hypoplasia has also been associated with congenital heart defects in children with and without Down syndrome; this is most probably caused by a disruption in early fetal development (46). In the present study, 23 (46%) children with Down syndrome had a congenital heart defect. Among the children with Down syndrome, no difference in DM/DD was found between those with or without congenital heart defects (*p* = 0.55).

### Optic Disc Insertion

The insertion of the optic nerve head in relation to the sclera was evaluated by the ovality, torsion or tilting of the disc, as well as by the presence of crescents. Typically, the optic nerve is slightly oval, with a perpendicular insertion onto the scleral canal. The photoreceptors, pigment epithelium, Bruch membrane and choroid terminate sharply at the optic disc margin (34). However, small differences between these openings can create a thin halo, the exact arrangement being specific to individual subjects (39). If the scleral canal is not oval, but has a D shape and is obliquely inserted onto the sclera, then it creates a crescent between the sclera and the retinal opening (34). These crescents can be scleral if the choroid does not reach the disc margin, or choroidal if only the pigment epithelium does not reach the disc margin. Temporal crescents are mostly found in myopic eyes and may be slowly progressive. Congenital crescents are located below the disc in the vast majority of cases, and most likely represent a failure of the embryonic fissure to close at the point where the optic stalk reaches the optic cup (39). The choroid, Bruch membrane and pigment epithelium terminate at various distances from the optic disc margin. In the present study, the optic nerve had a more variable shape (Figure 3) and was also more frequently oval, torted and tilted in children with Down syndrome than in controls. In line with these observations, we also observed an above- average prevalence of crescents in the children with Down syndrome (Figure 4). These findings may be explained by the higher prevalence of oblique astigmatism observed in these children, as both myopia and oblique astigmatism are associated with oval (48) and tilted optic discs (49, 50). Recently, subclinical keratoconus has been found to be common in patients with Down syndrome(51). This could explain the high prevalence of astigmatism, especially oblique, found in this study. The associated astigmatism, in the case of subclinical keratoconus, could, on one hand, produce an appearance of tilted disc. However, in this study, all eyes of children with Down syndrome and tilted disc had also a crescent. The crescent presence cannot be explained by an eventual keratoconus. On the other hand subclinical keratoconus could further explain the diminished visual acuity in children with Down syndrome.

Temporally located crescents were more frequently present in children with and without Down syndrome and myopia or myopic astigmatism (Figure 5). Crescents located below the disc were the most common type of crescents found in the children with Down syndrome and were present in eyes with all types of refraction, except myopia. Other forms of localization were rare (Figure 5). We found that more children with Down syndrome had peripapillary atrophy (Figures 4E,H, 6C) and pigment anomalies (Figure 6); this was probably a consequence of the abnormal insertion of the disc onto the sclera. Tilted optic discs, peripapillary atrophy and scleral crescents have also been described in subjects with Down syndrome in other studies (3, 20, 23). Larger proportions of myopic crescents (13.2%) and tilted discs (8.1%) were previously reported in a population of adults with Down syndrome, along with a high prevalence of myopia (24). As confirmed in this study, such anomalies do not represent conditions which could threaten the vision and are considered more as variants of the normal spectrum. This could probably be the reason why these anomalies are not mentioned in a larger number of studies. The high prevalence of such anomalies in children with Down syndrome, however, is of interest and should be investigated further.

### Other Optic Nerve Anomalies

In this study, four (8%) of the children with Down syndrome had optic disc drusen. Figures 7A,C shows the fundus images of two children with Down syndrome, while Figures 7B,D shows the corresponding B-scan ultrasonography.

Optic disc elevation, caused either by pseudo papilledema (16, 18, 23, 52) or true papilledema (15), has already been related to Down syndrome in the literature. It is therefore essential to differentiate pseudo papilledema from true papilledema in children with Down syndrome as some of their associated comorbidities can cause an increase in intracranial pressure (15).

Optic disc pallor was identified in only one child with Down syndrome in the present study, most likely representing a coincidental finding, similar to a few other cases reported previously (19, 20, 23). We did not identify any other sporadically described optic nerve anomalies in our subjects, such as morning glory (53, 54), optic nerve coloboma (55), optic nerve glioma (56), optic nerve atrophy (57) and optic nerve pit (24, 58).

### Visual Acuity

Most of the optic nerve anomalies identified in the children with Down syndrome in the present study represent developmental anatomic defects. None of these are specific to the syndrome itself, and multiple other non-specific ocular abnormalities have been previously described in the literature on Down syndrome. Most likely, these optic nerve abnormalities have no or little impact upon visual function. The present study confirmed that the visual acuity of children with Down syndrome is reduced when compared to their peers without Down syndrome (*p* = 0.001; Figure 8).

We analyzed some potential sources of reduced vision in the children with Down syndrome and attempted to determine whether optic nerve anomalies represent a potential cause. The fact that both groups had similar refraction problems, anisometropia and strabismus, and because there was comparable compliance in terms of wearing optical correction devices between the two groups, the risk of potential bias when comparing visual acuity was reduced.

Hypoaccommodation has been suggested to be a factor capable of reducing visual acuity in children with Down syndrome, predominantly by causing poor near focusing and bilateral amblyopia (59). In the present study, among children with Down syndrome, we found no significant difference in visual acuity between those with normal accommodation vs. hypoaccommodation (*p* = 0.10; Figure 9). The homogeneity of the visual acuity in both of these sub-groups was most probably due to the optical correction (bifocal or multifocal lenses) prescribed for these children, along with the excellent compliance regarding wearing the spectacles.

The fifteen (30%) children with Down syndrome and nystagmus had significantly reduced vision compared to children with Down syndrome without nystagmus (*p* = 0.0001). In these 15 cases, the nystagmus was always horizontal, either latent-manifest or manifest, and probably sensorial in those with high myopia (five children). Strabismus was an associated finding in seven subjects. Nevertheless, the visual acuity was still inferior in children with Down syndrome without nystagmus compared to the controls (Figure 10).

The size of the optic disc did not correlate with the globally reduced visual acuity, in the sense that the smallest optic nerves did not necessarily cause the poorest vision (*r* = 0.39, *p* = 0.31). Such a direct correlation between the size of the disc and the visual acuity is not possible in hypoplastic discs. Visual acuity ranged between normal vision to light perception, it is primarily determined by the integrity of the papillomacular nerve fiber bundle and not by the optic nerve size (60). Visual acuity was abnormal in eight (80%) of the ten children with Down syndrome who had a DM/DD >3.5 or a double ring sign. However, others factors could be responsible for the decrease of visual acuity in this cases.

Of the 13 children with Down syndrome who had a tilted disc, the vision was found to be abnormal in 11 (84%). Tilted discs are usually smaller and contain fewer axons, and overlaps between tilted and hypoplastic nerves are often present (61). Effects on visual acuity (50), visual field (32, 49) and color vision (62) are possible; however, in most cases, they are not sufficient to significantly reduce vision. The decreased visual acuity found in our children with Down syndrome and tilted disc is most probably multifactorial and does not represent a direct consequence of the anomalous disc.

We compared the visual acuity of eyes with and without crescents in children with Down syndrome. The presence of crescents did not have a significant influence on visual acuity (*r* = 0.35, *p* = 0.12).

The neurosensory deficits observed in patients with Down syndrome (lower performance on visual tests and VEP abnormalities) could not be explained by the optic nerve head anomalies described in this study. The origin of these deficits is more likely to be found on the visual pathways and not at the level of the optic nerve head.

### Study Limitations and Possible Bias

This study has several limitations, which need to be considered. First, the images were considered assessable and analyzed by a single observer. Interobserver variability regarding DM/DD assessment has been reported, especially in smaller discs (41). However, the DM/DD, cup-to-disc ratio and ovality assessments were performed and double-checked by the author. Indeed, a third measurement was acquired if the first two measurements were significantly different. Second, the precise classification of scleral and choroidal crescents, as well as differentiation from peripapillary atrophy, was based only on photographic images. This may have led to an inaccurate distinction between these features. Optical coherence tomography would help to increase accuracy and precisely determine the distance to the disc margin of each layer (choroid, Bruch membrane or pigment epithelium). Unfortunately, optical coherence tomography of the optic nerve was performed in only a select few of the patients. Third, visual field assessment could be of valuable help in determining optic nerve function, however, acquiring accurate visual field measurements in children with Down syndrome is rarely possible because of cooperation issues. Finally, the true impact of optic nerve anomalies on visual function should be explored in more depth; visual acuity is reduced by multiple factors, which may vary between individuals.

## Conclusion

The present study revealed a high prevalence of optic disc developmental anomalies in children with Down syndrome and analyzed possible causes for reduced visual acuity in these children. The novel findings of this study were corroborated with an extensive literature search on the subject.

The optic nerve heads were significantly smaller in these children. However, we did not identify a direct relationship between disc size and visual acuity in children with Down syndrome. Small discs, on the other hand, might be capable of promoting drusen formation (63). All optic nerve anomalies could potentially reduce visual acuity, depending upon their type and severity. Nevertheless, the precise reasons for abnormal visual acuity in children with Down syndrome must be considered on an individual basis, as multiple factors may be involved to varying degrees. These findings would allow a better knowledge of ocular problems in Down syndrome leading to improved ophthalmological management.

## Data Availability

All data are available upon request. For further information, please contact Lavinia Postolache at Lavinia.Postolache@ulb.ac.be.

## Ethics Statement

This study was carried out by the recommendations of the Declaration of Helsinki, Institutional Review Board, with written informed consent from all subjects. All subjects gave written informed consent. The protocol was approved by the Institutional Ethics Committee, of Queen Fabiola University Children's Hospital (CHE n°23/19).

## Author Contributions

LP was responsible for conception, design, analysis and interpretation of the data and wrote the article.

### Conflict of Interest Statement

The author declares that the research was conducted in the absence of any commercial or financial relationships that could be construed as a potential conflict of interest.
